# Correction to “Assessment of Chronic Rhinosinusitis With Nasal Polyps: Development of the Nasal Polyp Patient Assessment Scoring Sheet Tool”

**DOI:** 10.1002/lio2.70137

**Published:** 2025-05-24

S. Alsaleh, N. Kamal, C. Hopkins, et al., “Assessment of chronic rhinosinusitis with nasal polyps: development of the Nasal Polyp Patient Assessment Scoring tool,” *Laryngoscope Investig Otolaryngol* 9, no. 3 (2024): e1277, https://doi.org/10.1002/lio2.1277.

- **Figures** 2 and 3 are now updated with an additional reference (now reference 20). This is reflected in the section on SNOT‐22.

Reference 20 — National Comparative Audit of Surgery for Nasal Polyposis and Rhinosinusitis, Royal College of Surgeons of England. SINO‐NASAL OUTCOME TEST (SNOT‐22). https://bpb‐us‐w2.wpmucdn.com/sites.wustl.edu/dist/1/3538/files/2022/06/English_United‐States_S22_2019‐12‐24_Review‐Only.pdf. Accessed February 28, 2025.

A disclaimer stating “*All rights reserved. Copyright 2006. Washington University in St. Louis, Missouri*.” has been added to the SNOT‐22 tool to ensure compliance with the copyright clause of Washington University.

Link: https://otolaryngologyoutcomesresearch.wustl.edu/research/clinical‐research/sinusitus/sino‐nasal‐outcome‐tests‐snot/.

**FIGURE 2:**
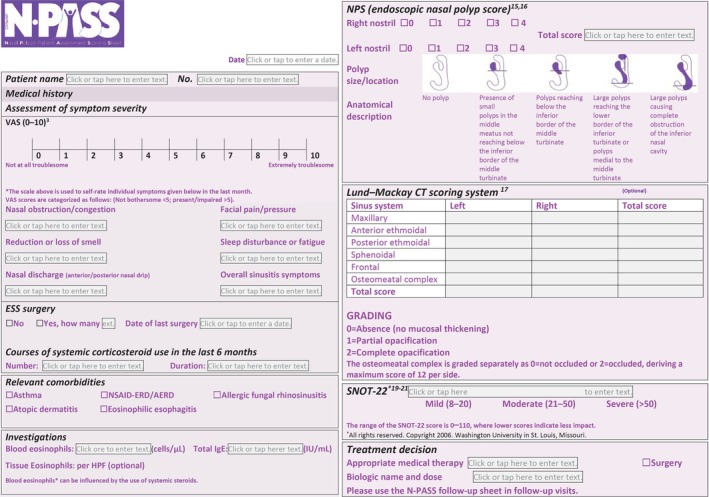
The N‐PASS tool. N‐PASS, Nasal Polyp Patient Assessment Scoring Sheet.

**FIGURE 3:**
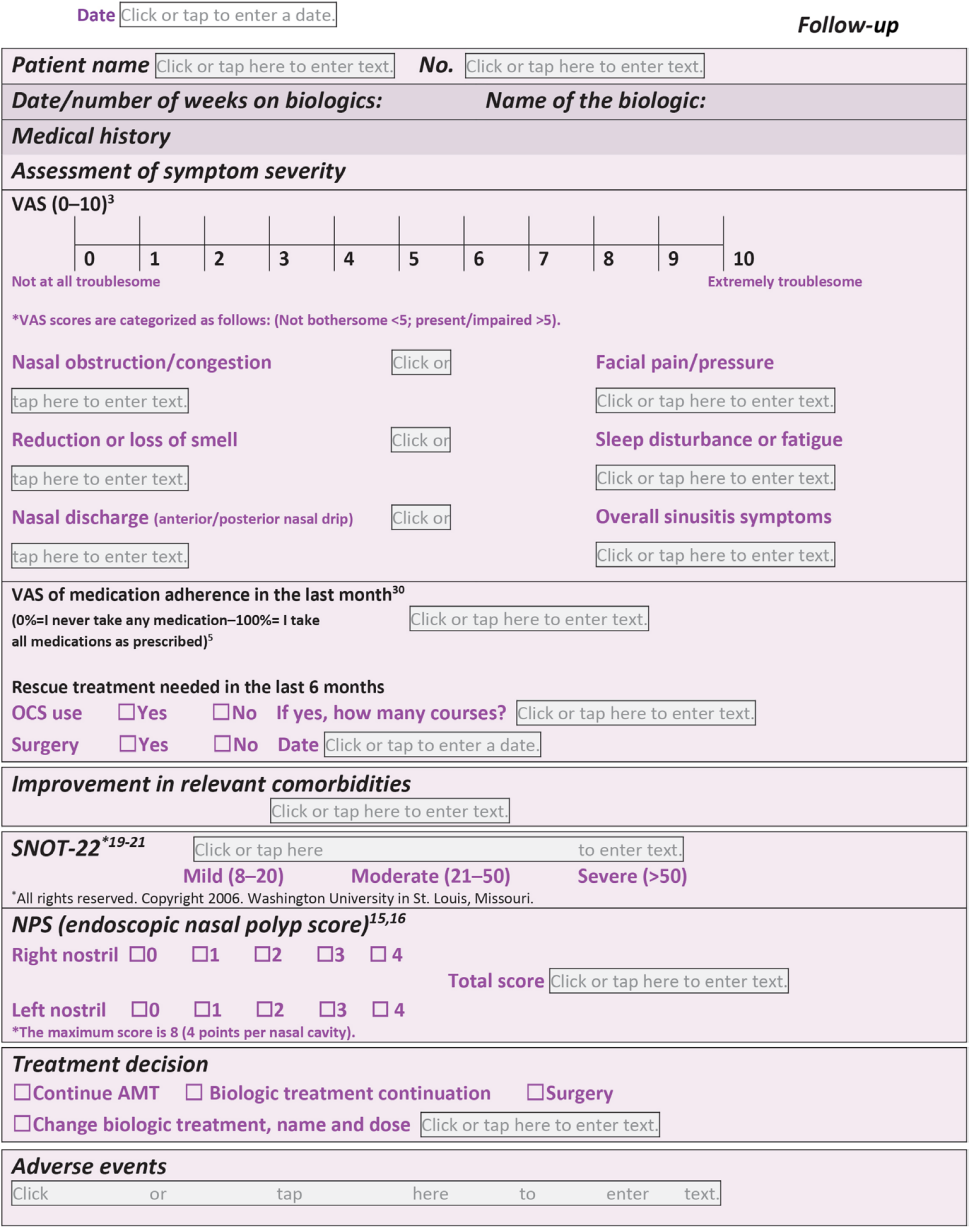
The N‐PASS follow up tool. N‐PASS, Nasal Polyp Patient Assessment Scoring Sheet.

**FIGURE 4:**
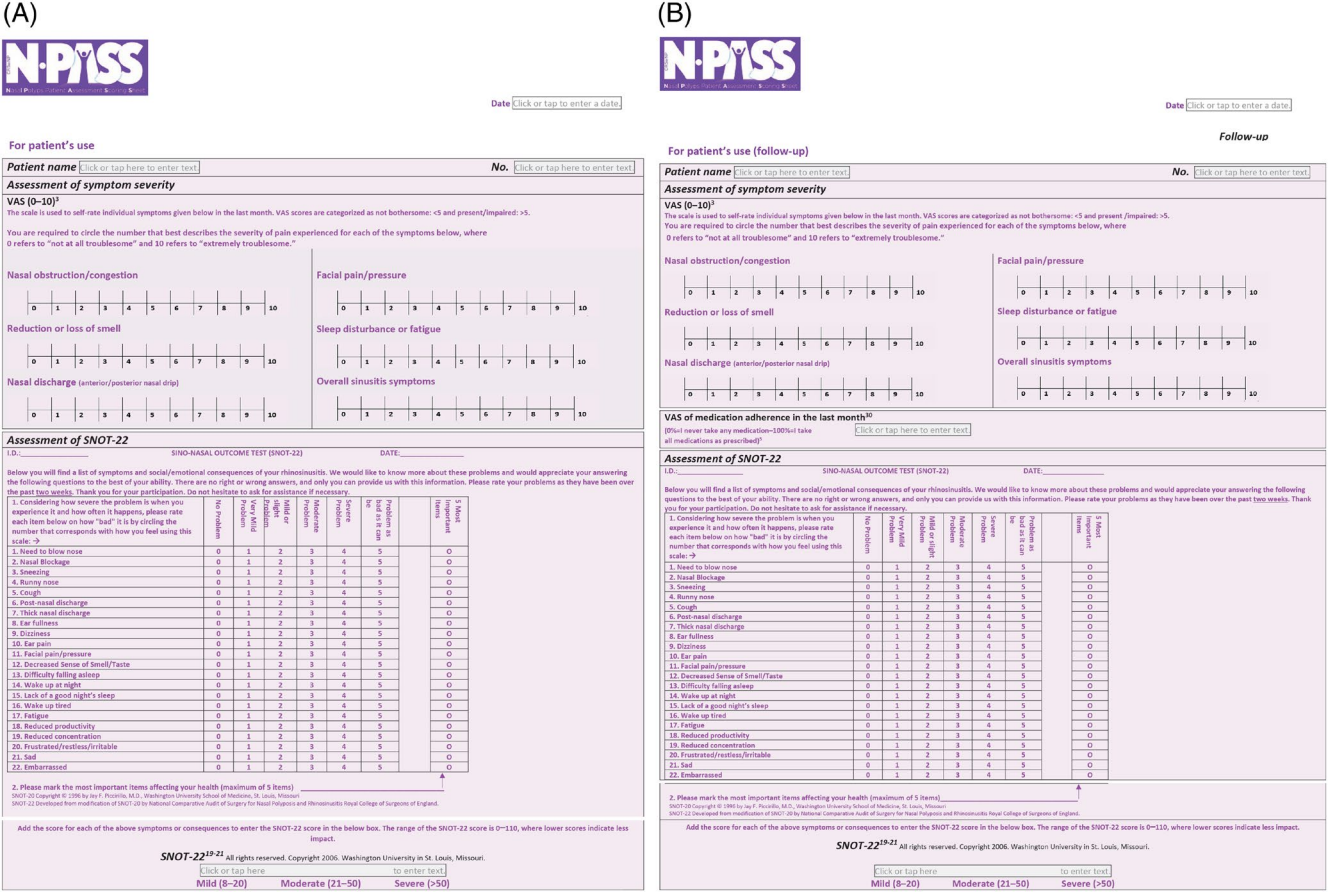
(A) The N‐PASS tool for patient’s response. (B) The N‐PASS follow up tool for patient’s response. N‐PASS, Nasal Polyp Patient Assessment Scoring Sheet.

- In **Figures** 4A and 4B, modifications were made to ensure resemblance to the SNOT‐22 tool. The figures were modified to appear as in the link provided (without modification or editing).
  This is to ensure complete compliance with the copyright notice of Washington University.
  Link: https://otolaryngologyoutcomesresearch.wustl.edu/research/clinical‐research/sinusitus/sino‐nasal‐outcome‐tests‐snot/Additionally, a new reference was added (now reference 20) to ensure comprehensiveness.
- Tables **1** and **2** have been updated under the SNOT‐22 domain. Two references have been added to ensure comprehensiveness: a new reference (now reference 20) and an update with the old reference (previously 20 and now updated to 21).

**TABLE 1:** Components of the N‐PASS evaluation sheet (high agreement parameters).

Domain evaluated	Components	Variables	Remarks
Medical history	A 10‐point self‐reported VAS^3^	Severity of symptoms: 0 = Not at all troublesome 10 = Extremely troublesome – Nasal congestion/obstruction – Nasal discharge (anterior/posterior nasal drip) – Reduction/loss of smell – Facial pain/pressure – Sleep disturbance or fatigue – Overall sinusitis symptoms	Grading: Not bothersome: < 5 Present/impaired: > 5
	ESS surgery	– Number – Date of last surgery	
	Courses of systemic corticosteroid in the last 6 months	– Number – Duration	
	Relevant comorbidities	– Allergic fungal rhinosinusitis – NSAID‐ERD/AERD – Asthma – Atopic dermatitis – Eosinophilic esophagitis	
Investigations	Laboratory evaluation	– Blood eosinophils – Total IgE – Tissue eosinophils (optional)	Blood eosinophils can be influenced by the use of systemic steroids.
Assessment of disease severity	Endoscopic NPS)^15,16^	– Polyp size	The maximum score is 8 (4 points per nasal cavity)
	Lund–Mackay CT Scoring System^17^	– Maxillary – Anterior ethmoidal – Posterior ethmoidal – Sphenoidal – Frontal – Osteomeatal complex	Grading of each sinus: 0 = Absence (no mucosal thickening) 1 = Partial opacification 2 = Complete opacification The osteomeatal complex is graded as follows: 0 = Not occluded 2 = Occluded, deriving a maximum score of 12 per side.
	SNOT‐22^19–21^	– 22 items	The range of the SNOT‐22 score is 0–110, where lower scores indicate less impact. The score is classified as follows: 8–20: Mild 21–50: Moderate > 50: Severe
Treatment decision		– AMT – Surgery – Biologic (name and dose)	Treatment is at the discretion of the physician. Physicians are encouraged to use the follow‐up sheet with all interventions.

Abbreviations: AMT: appropriate medical therapy; CT: computed tomography; ESS: endoscopic sinus surgery; IgE: immunoglobulin E; N‐PASS: nasal polyp patient assessment scoring sheet; NPS: nasal polyp score; NSAID‐ERD/AERD: nonsteroidal anti‐inflammatory drug‐exacerbated respiratory disease/aspirin‐exacerbated respiratory disease; SNOT: SinoNasal Outcome Test; VAS: visual analog scale.

**TABLE 2:** Components of the N‐PASS follow‐up sheet (high agreement parameters).

Domain evaluated	Components	Variables	Remarks
Medical history	If started on biologics:	Number of weeks on biologics Name of biologic used	
	A 10‐point self‐reported VAS^3^	Severity of symptoms: 0 = Not at all troublesome 10 = Extremely troublesome Nasal congestion/obstruction Nasal discharge (anterior/posterior nasal drip) Reduction/loss of smell Facial pain/pressure Sleep disturbance or fatigue Overall sinusitis symptoms	Grading: Not bothersome: < 5 Present/impaired: > 5
	VAS score of medication adherence in the last month^30^	0%: I never take medications 100%: I take all medications as prescribed	
	Rescue treatment needed in the last 6 months	Oral corticosteroids (yes/no) If yes, how many courses? Surgery (yes/no)	
	Improvement in relevant comorbidities	Allergic fungal rhinosinusitis NSAID‐ERD/AERD Asthma Atopic dermatitis Eosinophilic esophagitis	
Assessment of disease severity	Endoscopic NPS^15,16^	Polyp size	The maximum score is 8 (4 points per nasal cavity).
	SNOT‐22^19–21^	22 Items	The range of the SNOT‐22 score is 0–110, where lower scores indicate less impact. The score is classified as follows: – 8–20: Mild – 21–50: Moderate – > 50: Severe
Treatment	Decision	AMT Surgery Biologic: Continue or change to: ___	At the discretion of the treating physician.
	Adverse effects	Recording any possible complications or side effects that may occur during the treatment	

Abbreviations: AMT: appropriate medical treatment; CT: computed tomography; NPS: nasal polyp score; NSAID‐ERD/AERD: nonsteroidal anti‐inflammatory drug‐exacerbated respiratory disease/aspirin‐exacerbated respiratory disease; SNOT: SinoNasal Outcome Test; VAS: visual analog scale.

Reference 20 — National Comparative Audit of Surgery for Nasal Polyposis and Rhinosinusitis, Royal College of Surgeons of England. SINO‐NASAL OUTCOME TEST (SNOT‐22). https://bpb‐us‐w2.wpmucdn.com/sites.wustl.edu/dist/1/3538/files/2022/06/English_United‐States_S22_2019‐12‐24_Review‐Only.pdf. Accessed February 28, 2025.

Reference 21 (previously 20 and updated to 21) — Toma S, Hopkins C. Stratification of SNOT‐22 scores into mild, moderate or severe and relationship with other subjective instruments. *Rhinology*. 2016;54(2):129–133. doi:10.4193/Rhino15.072.

We apologize for these errors.

